# Lesbian and Gay Individual Parenting Desires in Heteronormative Contexts

**DOI:** 10.5964/ejop.v16i2.1808

**Published:** 2020-05-29

**Authors:** Diego Lasio, Jessica Lampis, Roberta Spiga, Francesco Serri

**Affiliations:** a Department of Pedagogy, Psychology, Philosophy of University of Cagliari, Cagliari, Italy; b Centro de Investigação e Intervenção Social, Instituto Universitário de Lisboa (ISCTE-IUL), Lisbon, Portugal; University of Wollongong, Wollongong, Australia

**Keywords:** LGBT, heteronormativity, parenting desire, parenting intention

## Abstract

The cultural, social and institutional barriers that LGBT (Lesbian, Gay, Bisexual, Transgender) individuals have to face play crucial roles in their desires and intentions to have children. However, unlike the many studies on the decision-making process in the transition to parenthood, few studies have analysed the origins of parenting desires and intentions among LGBT individuals. This study explores the desires and intentions to have children amongst a sample of childless lesbian and gay Italian individuals. A sample of 285 participants (127 women and 158 men) completed a research protocol composed of items evaluating the strength of their desire to have children, their intentions about having children and their general attitudes towards parenting. The findings revealed how, despite the persisting depth of heteronormativity in the country and the absence of legal protection for lesbian and gay parents, a large percentage of participants expressed the desire and intention to have a child. These parenting intentions would seem to be positively influenced mainly by the negative attitudes towards childlessness and by the value attributed to parenthood.

Although new discourses on families have emerged since the 1980s (Weston, 1991), and the process of “homonormalization” (Roseneil, Crowhurst, Hellesund, Santos, & Stoilova, 2013) has contributed to challenging the notion of “the family” as a unique and fixed object, many individuals are still denied access to kinship due to their non-compliance with the heteronormative model of family. Gay and lesbian couples constitute one of the most widely debated cases of exclusion from “the family”, both because of the growing number of same-sex couples who do decide to have children and because of the political pressures for their legal recognition applied by LGBT (Lesbian, Gay, Bisexual, Transgender) social movements and supra-national institutions, such as the EU (Santos, 2004, 2013). Although their rights are not always recognized, lesbian and gay families exist throughout Europe and the United States, with a level of incidence that makes no longer possible to consider them as a silent minority. In the USA, for example, ever since the early 2000s, the National Survey of Family Growth (Chandra, Martinez, Mosher, Abma, & Jones, 2005) revealed that, although LGBT individuals were still less likely than heterosexual individuals to have children (Gates, Badgett, Macomber, & Chambers, 2007), 35% of lesbian women and 16% of gay men had either biological or adopted children. Furthermore, about 23% of lesbian and bisexual women reported caring for someone else’s child, compared to 12% of heterosexual women. However, despite numerous studies (e.g. Biblarz & Stacey, 2010; Carneiro, Tasker, Salinas-Quiroz, Leal, & Costa, 2017; Fedewa, Black, & Ahn, 2015; Tasker, 2010) having demonstrated no relationship between the wellbeing of children and the sexual orientation of their parents, negative beliefs about lesbian and gay parenthood still remain very widespread (e.g. Baiocco, Nardelli, Pezzuti, & Lingiardi, 2013; Costa, Pereira, & Leal, 2019; Gato & Fontaine, 2016; Lingiardi et al., 2016).

As regards the legislative landscape, most western countries have now recognized the rights of same-sex couples, with legal recognition of lesbian and gay parenthood also gradually increasing. Nowadays, the majority of EU countries have detailed regulations concerning same-sex marriages or civil unions and gay and lesbian parenthood: Denmark, for instance, was the first to introduce a civil union register, which included same-sex couples in 1999, and the first to allow adoption for non-married persons regardless of their sexual orientation. Moreover, since 2007, Danish lesbian women have also received access to assisted reproduction technologies. In the late 1980s and during the 1990s, many Northern European countries, such as Sweden, Iceland and the Netherlands followed suit, with different forms of regulation of *more uxorio* cohabitation, marriage, civil union and gay and lesbian parenthood. In the 2000s, such principles spread throughout the whole of Europe: countries including France, the United Kingdom and Germany recognized same-sex partnerships and in some cases gay and lesbian couples have been allowed to adopt and to access reproductive technologies.

With regard to the countries where the legacy of Catholicism remains more significant, Spain was the first to legalize gay and lesbian civil marriages. Despite the Catholic Church campaigning heavily against same-sex marriage in the country, the Parliament amended the Civil Code, rendering same-sex civil marriages equal to heterosexual relationships and extending the right to have children to gay and lesbian couples by any form of adoption or assisted reproductive technologies, including in vitro fertilization but not surrogacy. Portugal, a country where the Catholic Church has long played a dominant role in defining “what is socially desirable and what is morally wrong” (Santos, 2004, p. 168), was the eighth country in the world to legalize same-sex marriage in 2010, which followed the recognition of same-sex couples to the right of civil partnership ten years before. In this country, same-sex marriage was approved with the provision that lesbian and gay couples could neither adopt children nor access reproductive technologies (Brandão & Machado, 2012), but the Portuguese Parliament overturned the bans on parenting and allowed adoption and assisted reproduction by same-sex couples and single women in February 2016. However, such legislative progress did not bring to an end heteronormativity nor even to the multiple forms of discrimination in the public sphere (de Oliveira, Lopes, Cameira, & Nogueira, 2014).

Within this framework, Italy presents specific features and peculiarities because until May 2016 it was one of the few European countries where same-sex couples received no legal recognition. This legislative gap was partially filled through the approval of law 76/2016 (Gazzetta Ufficiale, 2016), which recognized same-sex civil unions and extended to them most of the rights of married heterosexual couples, such as the right to receive a widow(er)’s pension and to inherit each other’s assets. However, the law has also reinforced the distinction between heterosexual and non-heterosexual couples by defining same-sex civil unions as ‘specific social formations’, and excluding the duty of fidelity between their partners because, according to opponents, it would have made civil unions too similar to traditional marriage (Lasio & Serri, 2019). The three-year long debate which led to the approval of the law featured strong opposition both inside and outside parliament as well as sustained opposition by civil society organizations, Vatican hierarchies, and citizen movements informed by Catholic thought (Lasio, Congiargiu, De Simone, & Serri, 2019; Lasio & Serri, 2019). The nodal point around which the different and opposing positions were built up was section number 5 of the draft bill, which would recognize the right of one partner to adopt the biological children of the other partner by introducing the so-called *step-child adoption*. This legal stipulation on adoption rights proved so controversial that it led to deep divisions in the government majority and, as a result, had to be deleted in order for the law to pass, thus denying many children growing up in families with same-sex parents the same legal protection as their peers (Lampis, De Simone, Fenu, & Muggianu, 2017).

The absence of legal protection for same-sex parents and their children affects a large number of individuals who live in the country: according to the fifteenth census of the Italian National Institute of Statistics (ISTAT, 2012), there were 7,513 same-sex couples in Italy totalled and with 529 raising children. As the Institute itself pointed out, these data might represent an underestimate not only because many couples have preferred not to come out but also due to the census referring only to cohabiting couples (Baiamonte & Bastianoni, 2015). The data nevertheless reveal that same-sex couples and lesbian and gay parenthood are a well-established reality in the country even while heteronormativity (Kitzinger, 2005; Warner, 1991) still vetoes any expression of nonconventional sexualities and makes heterosexual reproduction and kinship appear as the obvious choice, while lesbian and gay couples remain alienated from kinship (Weston, 1991).

The abovementioned opposition to the recognition of same-sex couples and their children testify to the persisting depth of heteronormativity in the country, and contribute towards perpetuating homonegativity, stigmatization and discrimination (e.g. Lingiardi, Falanga, & D’Augelli, 2005; Lingiardi et al., 2016; Pistella, Tanzilli, Ioverno, Lingiardi, & Baiocco, 2018). According to the European Union Agency for Fundamental Rights (2012), the percentage of Italian people who believe that gay and lesbian parenthood harms children stands at among the highest in Europe. As many authors (Baiocco et al., 2013; Bertone, 2017; Bertone & Franchi, 2014) have argued, these prejudices also persist because of religious and cultural traditions that encourage homophobic opinions and sentiments. Different studies identified how a conservative political orientation and deep religious involvement are related with negative attitudes towards lesbian and gay marriage and parenthood (Averett, Strong-Blakeney, Nalavany, & Ryan, 2011; Baiocco et al., 2013; Costa et al., 2014; D’Amore, Green, Scali, Liberati, & Haxhe, 2014; Gross, Vecho, Gratton, D’Amore, & Green, 2018; Olson, Cadge, & Harrison, 2006).

Hegemonic heteronormative norms maintain LGBT individuals in vulnerable positions, which bear evident effects in terms of citizenship, rights and social prejudices (Lopes, de Oliveira, Nogueira, & Grave, 2017). Furthermore, institutionalized heteronormativity wields negative influences on the self-beliefs of LGBT individuals, who can correspondingly manifest negative attitudes toward themselves and their parental competences (e.g. Baiocco, Argalia, & Laghi, 2014; Lingiardi, Baiocco, & Nardelli, 2012; Pacilli, Taurino, Jost, & Van der Toorn, 2011). Heteronormativity may thus be upheld by LGBT individuals who might perceive transgressions of the heteronorms as a cost and a personal risk (de Oliveira, Costa, & Nogueira, 2013), and negative religious-based attitudes are therefore susceptible to internalization by lesbian, gay and bisexual individuals who effectively end up endorsing religious arguments that they are not fit to be parents (Baiocco et al., 2014). A recent Portuguese study (Costa & Bidell, 2017), for example, found that religiosity negatively predicted lesbian and gay individuals’ intentions over raising children. The collusion of LGBT individuals with the (hetero)norms about gender, sexuality, reproduction and kinship, together with the absence of legal recognition and the negative social attitudes toward non-heterosexual parenthood, contribute to maintain LGBT individuals exiled from kinship (Lasio, Serri, Ibba, & de Oliveira, 2019).

In the Italian context, gay and lesbian individuals sometimes share the same negative attitude as heterosexual individuals as regards the parental competences of same-sex couples (Pacilli et al., 2011). Recent research (Lasio, Serri, Ibba, & de Oliveira, 2019) has highlighted that the pervasiveness of hegemonic heteronormativity is so deep reaching that LGBT activists might collude in some respects with the cultural assumptions that sustain the premises of their subjugation. Moreover, heteronormativity in Italy prevents sexual minorities from expressing themselves even in social contexts that, on the one hand, are involved in combatting social exclusion and, on the other hand, that deny any marginalization of LGBT employees or consider this a problem these employees have to solve themselves (Priola, Lasio, De Simone, & Serri, 2014; Priola, Lasio, Serri, & De Simone, 2018).

The cultural, social and institutional barriers that LGBT individuals face all play crucial roles in their desires and intentions around raising children; however, unlike the many studies on decision-making processes in the transition to parenthood (Goldberg, 2010), few studies have analysed the origins of parenting desires and intentions among LGBT individuals. Bos, Van Balen, and Van den Boom (2003) identified three main facets to understanding the desires and intentions of becoming parents that focused on the importance parenting assumed for women. According to a first perspective, grounded on a psychoanalytic point of view, motherhood plays an essential role in the development of female identities. A second perspective, supported by feminist researchers, claims that the desire to have children arises out of the social pressures that constrain women in both body and motherhood (Amâncio & de Oliveira, 2006), while also highlighting how the traditional view of motherhood constitutes an obstacle to the personal development of women (De Simone, Lasio, Onnis, & Putzu, 2017; Lasio, Putzu, Serri, & De Simone, 2017; Lasio, Serri, De Simone, & Putzu, 2013). A third branch of research studies parenthood according to a cost-benefit model: mothers decide whether or not to have children by evaluating the advantages and disadvantages of parenthood. According to Bos et al. (2003), this evaluation holds no influence on the desire for the first child but might weigh on the decision to have further children.

While these perspectives may generate insights into the understanding of heterosexual couple desires and intentions to have children, they lack any scope for understanding the parenting desires of LGBT individuals. Although from the 1990s onwards there has been broad acceptance that lesbian and gay individuals would like to become parents, only few studies have analysed the origins of parenting desires and intentions among LGBT individuals. In Sbordone’s (1993) study, for a sample of gay men, about half of the participants expressed the desire to have children. Moreover, no differences were found between those men who were already fathers and those who were not. In the United States, research findings (D’Augelli, Rendina, Sinclair, & Grossman, 2007) identified how LGBT youths between the ages of 16 and 22 held high expectations and desires about parenthood: in a sample of 133 lesbian and gay individuals, only 14% reported not being interested in becoming parents. This percentage perfectly matches the data from the National Survey of Family Growth on heterosexual women: only 14% declared a lack of desire for parenthood (Chandra, Martinez, Mosher, Abma, & Jones, 2005).

Two other research teams (Gates et al., 2007; Riskind & Patterson, 2010) analysed data from the 2002 National Family Growth Survey with the aim of studying parenting intentions, desires, and attitudes among LGBT individuals in the United States. Participants were aged from 15 to 44 years with both studies highlighting how LGBT individuals might be less likely than heterosexual individuals to state a desire to have children but also revealing how half of gay men and lesbian women held the desire to have children while furthermore endorsing the value of parenthood just as strongly as heterosexual participants (Riskind & Patterson, 2010).

Riskind and Patterson (2010) also inquired whether participants really *intended* to have children. Among those expressing parenting desires, 67% of gay men and 83% of lesbian women stated an intention to have a child versus 90% of heterosexual men and 72% of heterosexual women. Globally, 30% of gay men and 33% of lesbian women expressed both parenting desires and intentions. Consistent with previous findings (Gates et al., 2007), the gap between parenting desires and intentions was larger for gay men than for their heterosexual counterparts even when controlling for variables such as participant age, ethnicity and education. Research also demonstrated how women had stronger desires to parent than men regardless of their sexual orientation (Bos et al., 2003; D’Augelli et al., 2007; van Balen & Trimbos-Kemper, 1995).

There is little research about parenting desires and intentions in same-sex couples outside of the United States but the existing research confirm the U.S. based findings. Bos et al. (2003), for example, studied the desires and motivations to have children in a sample of 100 lesbian couples and 100 infertile heterosexual couples, all attending a fertility clinic in the Netherlands. According to these authors, lesbian mothers spent more time thinking about the reasons for having children than heterosexual parents. In addition, lesbian biological mothers expressed stronger desires for parenthood than heterosexual mothers with the same also applying to lesbian social mothers when compared to heterosexual fathers. Rossi, Todaro, Torre, and Simonelli (2010) also reported a strong desire to have children in their exploratory study of an Italian sample of 226 lesbian and gay individuals: 57% of participants (61.4% women and 53.8% men) stated they wished to have children. This applied particularly to persons in relationships (61% expressing a desire for parenthood versus 39% of single individuals). Among those who expressed the desire to have children, 68% of gay men and 84.3% of lesbian women also stated the intention to advance with childbearing.

Baiocco et al. (2013) studied parenting desires and intentions in a sample of Italian gay men and lesbian women aged between 18 and 35. They reported that 51.8% of gay men and 60.7% of lesbian women declared parenting desires and that 30.2% of gay men and 46.3% of lesbian women expressed parenting intentions. Lesbian and gay individuals here reported significantly lower parenting desires and intentions than their heterosexual counterparts with lesbian women reporting higher desires and intentions when compared with gay men. This study also identified lesbian women as reporting stronger parenting desires than gay men. Both lesbian and gay individuals reported a higher gap between desires and intentions than heterosexual individuals. Finally, Scandurra and colleagues (2019) explored, in a sample of 290 Italian childless lesbian and gay individuals the influence of minority stress, gender differences, and legalization of civil unions in Italy on parenting desire and intention. The results indicated that the minority stressors associated with parenting dimensions partly explained the intention and desire to become parents, while perceived support from family or significant others buffered the effects of minority stressors on parenting dimensions. Furthermore, they found that lesbian women showed higher levels of parenting desire and intention than gay men and the levels of these parenting dimensions increased after the law on civil unions was enacted.

Both the Riskind and Patterson (2010) and Rossi and colleagues (2010) studies also approached participant attitudes toward parenting. Their results showed that homosexual individuals endorsed the values of parenthood as strongly as their heterosexual peers. Consequently, attitudes toward parenting were not susceptible to consideration as the reason gay and lesbian people were less likely to have children.

Despite the strength of religious values in Italy, we are not aware of any published research that considers the role of religious belief in the desire and intention to have children amongst lesbian and gay individuals. In addition to the study of Rossi and colleagues (2010), we have found only one other study that analysed attitudes toward parenting among Italian gay and lesbian people (Petruccelli, Baiocco, Ioverno, Pistella, & D’Urso, 2015). This research reported that left wing political preferences correlated with positive attitudes towards lesbian and gay parenthood. However, contrary to researchers’ expectations, no significant correlations were identified between religious involvement and attitudes toward same-sex parenthood.

## Study Aims

Starting out from these premises, this research project explores the parenting intentions and desires of childless lesbian and gay Italian individuals. Specifically, in keeping with previous studies, we assume that:

1. lesbian women report stronger parenting intentions than gay men
2. there is a gap between the parenting desires and the intentions of gay and lesbian individuals
3. individual variables (age, relationship status, religiosity, political positioning), general attitudes toward parenting and ideological positioning (agreement to the legal recognition of same-sex civil unions, marriage between same-sex individuals and adoption of children by same-sex couples) affect and shape parenting desires and intentions.

## Method

### Procedure and Participants

The study took place through an online survey. The platform Google forms was used to send the questionnaires to Italian associations politically active in combating discrimination based on sexual orientation and gender identity, with the invitation to forward them to their members and supporters. The inclusion criteria required potential participants to identify as either lesbian or gay, be aged over 18, and without any children. Participants gave their consent to study participation on the first page of the survey instrument. A demographic questionnaire was completed on the next survey page following receipt of the participant’s consent.

The final sample of 285 participants consisted of 127 women (44.6%) and 158 men (55.4%), with their ages ranging from 18 to 48 (*M* = 28.36 years, *SD* = 7.5). As regards their educational level, 7.4% held PhD degrees, 46% master’s or undergraduate degrees with 38.9% having graduated from high school, 2.1% with middle school certificates, and 5.6% with primary school certificates. 53.3% of the respondents were single, 22.5% cohabited and with 20.7% in stable relationships although neither married nor cohabiting. The relationship durations ranged from 1 to 32 years (*M* = 2.3 years, *SD* = 3). Table 1 presents the full sociodemographic characteristics of participants before Table 2 sets out their ideological positionings as regards same-sex couple civil unions, marriage between same-sex individuals and the adoption of children by same-sex individuals.

**Table 1:** Sample Characteristics (N = 285)

Characteristic	*n*	%
Sexual orientation		
Gay	158	55.4%
Lesbian	127	44.6%
Education		
Below high school	22	7.7%
High school diploma	111	38.9%
University degree	131	46%
PhD degree	21	7.4%
Religious beliefs		
Catholic	66	28.5%
Buddhism	12	3.5%
Eastern religion	9	2.5%
Atheist	198	69.5%
Religious practice		
Assiduous	9	3.2%
Frequent	13	4.6%
Occasional	59	20.7%
Never	204	76%
Political positioning		
Left	80	28.1%
Centre-left	149	52.1%
Centre	37	13%
Centre right	17	6.1%
Right	2	0.7%
Relationship status		
Single	152	53.3%
Cohabiting	74	22.5%
Partner	59	20.7%

**Table 2:** Ideological Positioning (N = 285)

Question	*n*	%
How much do you agree with the civil recognition of homosexual unions?		
Disagree	2	0.7%
Neither agree nor disagree	16	5.6%
Agree	158	93.7%
How much do you agree with marriage between same sex individuals?		
Disagree	7	2.5%
Neither agree nor disagree	13	4.6%
Agree	265	93%
How much do you agree with allowing homosexual individuals to adopt children?		
Disagree	11	3.9%
Neither agree nor disagree	9	3.2%
Agree	265	93%

### Measures

#### Individual and Demographic Characteristics

Participants were asked specific questions about their demographic and personal characteristics, including age, gender, sexual orientation, education, professional status, nationality, relationship status, religiosity, political positioning (Left, Centre-left, Centre, Centre-right, Right) and ideological positioning (agreement to the legal recognition of same-sex civil unions, marriage between same-sex individuals and adoption of children by same-sex couples).

#### Desire and Strength of Desire to Have Children

To measure the desire to have children, participants were asked to answer the item “Looking to the future, if it were possible, would you, yourself, want to have a baby at some time?” (Yes/No) (Riskind & Patterson, 2010). To evaluate the strength of this parenting desire (intensity of desire) we asked participants to answer the question “What are you willing to give up in order to have children?” (1 = it doesn't really matter to me, 6 = more than anything) (Bos et al. 2003).

#### Intentions and Reflections

To measure parenting intentions, participants were then asked: “Sometimes what people want and what they intend are different because they are not able to do what they want. Looking to the future, do you, yourself, intend to have a baby at some time?” (Yes/No) (Riskind & Patterson, 2010). To measure the thought process (reflection) involved in the process of deciding to have children, we asked “how often have you thought about the reasons for having children?” (1 = never, 3 = often).

#### General Attitudes Toward Parenting

We explored attitudes toward childlessness with the item “If it turns out that you do not have any children, would that bother you a great deal, somewhat, a little, or not at all?” (1 = not at all, 4 = a great deal) (Riskind & Patterson, 2010) before measuring the value of parenthood with the item “The rewards of being a parent are worth it, despite the cost and the work involved” (1 = Strongly disagree, 5 = Strongly agree) (Riskind & Patterson, 2010).

## Results

### Hypotheses

#### H1- Gender Differences in Parenting Desires and Intentions

84.6% of participants (M = 84.8%, F = 84.3%) reported parenting desires and 64.6% (M = 62%, F = 67.7%) reported their specific intention to have a child. The chi-squared test and ANOVA served to assess gender differences for these variables. The χ^2^ test did not reveal any significant differences between gay and lesbian individuals about parenting desire (χ^2^ = 0.017, *p* > .5) and parenting intention (χ^2^ = 0.997, *p* > .5). Additionally, the Anova results did not reveal any univariate effect of gender either on the strength of parenting desire, *F*(1, 283) = 0.48, *p* > .5, or on reflexivity, *F*(1, 283) = 0.97, *p* > .5.

#### H2- Discrepancy Between Parenting Desires and Intentions

The χ^2^ test revealed a significant difference between parenting desire and parenting intention (χ^2^ = 81.9, *df =* 1, *p* < .001). The desire to become parent was significant higher of the intention to have children. This trend was present both in the female (χ^2^ = 42.71, *df =* 1, *p* < .001) and in the male subsample (χ^2^ = 40.22, *df =* 1, *p* < .001).

#### H3- The Predictors of Parenting Desire and Intention

Two binomial logistic regressions (one for the desire dimension and the other for intention) and two hierarchical linear multiple regression analysis (one for the strength of desire and the other for reflexivity in relation to the intension dimension) were conducted to test our hypothesis and to analyse which of the variables considered shaped and affected parenting desires and intentions. The predictor variables included into the regression equation were age, gender, relational status, religiosity, political positioning, general attitudes toward parenting and ideological positioning with regard to same-sex civil unions, marriage between same-sex individuals and the adoption of children by same-sex individuals. There were neither correlations above .80 nor were there problems with multicollinearity (Variance Inflation Factor results of < 2.5 for each independent variable) in the regression analyses.

### Parenting Desire

The results conveyed how the model adapted well to the data from the first block of analysis. The appropriateness of the model reflected across the *fit index value* results (Omnibus Tests χ^2^ = 113.864, *df* = 11, *p* > .001; Hosmer and Lemeshow Test χ^2^ = 14.402, *df* = 8, *p* > .05, -2 log Likelihood = 131.038; Cox & Snell *R*^2^ = .330; Nagelkerke *R*^2^ = .572).

The odds ratio, measured for each of the predictor factors, referred to the increase or decrease in the probability of the subjects falling into two groups.

This correspondingly found that by increasing by one unit in the attitudes toward childlessness (*B* = -1.570; *OR* = 0.208; *p* < .001), the value of parenthood (*B* = -.997; *OR* = 0.369; *p* < .001) and the level of agreement with recognition of the opportunity to adopt children (*B* = -1.004; *OR* = 0.336; *p* < .001), the probability of subjects falling into the “desire” group increased.

Sexual orientation, age, religiosity and political positioning did not produce any significant effects (Table 3).

**Table 3:** Logistic Regression Model Predicting the Parenting Desire (yes = 0, no = 1)

Variable	*B*	*ES*	Wald(*df* = 1)	*OR*	95% CI for *OR*	
					*LL*	*UL*
Gender	0.734	0.521	1.984	2.084	0.750	5.788
Age	-0.001	0.031	0.001	0.999	0.941	1.061
Relational status	-0.019	0.217	0.008	0.981	0.641	1.502
Religiosity	-0.545	0.770	0.501	0.580	0.128	2.624
Religious practice	-0.307	0.461	0.443	0.736	0.298	1.815
Political positioning	-0.049	0.168	0.084	0.953	0.685	1.325
Attitudes toward childlessness	-1.570	0.326	23.261*	0.208	0.110	0.394
Value of parenthood	-0.997	0.240	17.227*	0.369	0.231	0.591
How much do you agree with the civil recognition of homosexual unions?	1.375	1.250	1.210	3.956	0.341	45.849
How much do you agree with marriage between same sex individuals?	0.993	0.687	2.088	2.699	0.702	10.374
How much do you agree with recognition of the opportunity for homosexual people, to adopt children?	-1.004	0.364	7.607*	0.366	0.180	0.748

*Note. OR =* odds ratio*;* CI *=* confidence interval.

*p < .01.

### Strength of Parenting Desire

The predictor variables were entered into the regression equation in three blocks by using the stepwise conditional method. In the first step (Model 1) we inserted the socio-demographic variables (gender, age, relational status, religiosity, religious practice an political positioning), in the second step (Model 2) we added, as predictors, the psychological variables (attitudes toward childlessness and value of parenthood), finally in the third step (Model 3) we added, as predictor, ideological positioning variables (How much do you agree with marriage between same sex individuals? How much do you agree with recognition of the opportunity for homosexual people, to adopt children?).

Model 3 was significant (*F* = 18.15, *p* < .001). The total proportion of variance in the strength of the parenting desire explained by all of the independent variables was 37.7% (*R*^2^ adjusted = .377). According to the standardized regression coefficient, attitudes toward childlessness (β = .472, *p* < .001) and the value of parenthood (β = .233, *p* < .001) significantly related to the strength of the parenting desire (Table 4).

**Table 4:** Hierarchical Linear Multiple Regression Model Predicting the Strength of Parenting Desire

MODEL 3- Variable	*B*	*SE*	β	*t*	*p*	VIF
Gender	.010	.102	.005	.099	.922	1.094
Age	-.006	.007	-.046	-.925	.356	1.144
Relational status	.079	.047	.081	1.686	.093	1.066
Religiosity	-.047	.149	-.021	-.312	.755	2.014
Religious practice	.068	.093	.048	.733	.464	1.933
Political positioning	.013	.032	.020	.408	.684	1.151
Attitudes toward childlessness	.465	.056	.462	8.296	< .001	1.422
Value of parenthood	.224	.053	.226	4.194	< .001	1.327
How much do you agree with the civil recognition of homosexual unions?	-.096	.142	-.040	-.677	.499	1.573
How much do you agree with marriage between same sex individuals?	-.049	.083	-.034	-.588	.557	1.506
How much do you agree with recognition of the opportunity for homosexual people, to adopt children?	.148	.084	.110	1.755	.080	1.815

### Parenting Intentions

The results demonstrated that the model adapted well to the data from the first block of analysis. The appropriateness of the model also reflected in the *fit index value* results (Omnibus Tests χ^2^ = 113.134, *df* = 11, *p* > .001; Hosmer and Lemeshow Test χ^2^ = 8.265, *df* = 8, *p* > .05; -2 log Likelihood = 232.557; Cox & Snell *R*^2^ = .383; Nagelkerke *R*^2^ = .526).

These results also identified how one unit increase in the attitudes toward childlessness (*B* = -1.216; *OR* = 0.296; *p* < .001), the value of parenthood (*B* = -0.777; *OR* = 0.460; *p* < .001), the level of agreement with the recognition of same-sex marriage (*B* = -0.681; *OR* = 0.506; *p* < .05) and the level of agreement with the opportunity for LGBT adoption of child (*B* = -0.846; *OR* = 0.429; *p* < .05) increased the probability of subjects falling into the “intention” group. The results also demonstrated that one unit increase in the age (*B* = 0.083; *OR* = 1.087; *p* < .001) raised the probability of subjects falling into the “no intention” group. Sexual orientation, religiosity and political positioning did not return any significant effects (Table 5).

**Table 5:** Logistic Regression Model Predicting the Parenting Intention (yes = 0, no = 1)

Variable	*B*	*ES*	Wald(*df* = 1)	*OR*	95% CI for *OR*	
					*LL*	*UL*
Gender	0.211	0.347	0.370	1.235	0.626	2.436
Age	0.083	0.024	12.343**	1.087	1.037	1.138
Relational status	0.066	0.156	0.178	1.068	0.787	1.450
Religiosity	-0.941	0.510	3.402	0.390	0.144	1.061
Religious practice	0.116	0.316	0.134	1.123	0.604	2.087
Political positioning	-0.031	0.114	0.076	0.969	0.775	1.212
Attitudes toward childlessness	-1.216	0.219	30.700**	0.296	0.193	0.456
Value of parenthood	-0.777	0.194	16.066**	0.460	0.314	0.672
How much do you agree with the civil recognition of homosexual unions?	0.865	0.441	3.840*	2.375	1.000	5.643
How much do you agree with marriage between same sex individuals?	-0.681	0.257	7.044*	0.506	0.306	0.837
How much do you agree with recognition of the opportunity for homosexual people, to adopt children?	-0.846	0.289	8.583*	0.429	0.244	0.756

*Note. OR =* odds ratio*;* CI *=* confidence interval.

*p < .05. **p < .01.

### Reflexivity Respect Parenting Intentions

The predictor variables were entered into the regression equation in three blocks by using the stepwise conditional method. In the first step (Model 1) we inserted the socio-demographic variables (gender, age, relational status, religiosity, religious practice an political positioning), in the second step (Model 2) we added, as predictors, the psychological variables (attitudes toward childlessness and value of parenthood), finally in the third step (Model 3) we added, as predictor, ideological positioning variables (How much do you agree with marriage between same sex individuals? How much do you agree with recognition of the opportunity for homosexual people, to adopt children?)

Model 3 was significant (*F* = 16.007, *p* < .001). The total proportion of variance in the reflexivity respect parenting intentions explained by all of the independent variables was 36.8% (*R*^2^ adjusted = .368). According to the standardized regression coefficient, attitudes toward childlessness (β = .472, *p* < .001) and the value of parenthood (β = .233, *p* < .001) significantly related to the strength of the parenting desire (Table 6).

Our analytical findings also revealed how older participants, who would regret not having children and who valued parenthood, reported greater levels of reflexivity as regards parenting intentions.

**Table 6:** Hierarchical Linear Multiple Regression Model Predicting Reflexivity With Respect to Parenting Intentions

MODEL 3 -Variable	*B*	*SE*	β	*t*	*p*	VIF
Gender	.054	.088	.031	.619	.537	1.094
Age	.014	.006	.116	2.290	.023	1.144
Relational status	-.009	.040	-.010	-.212	.832	1.066
Religiosity	.071	.129	.037	.552	.581	2.014
Religious practice	-.097	.080	-.079	-1.203	.230	1.933
Political positioning	-.038	.028	-.069	-1.364	.174	1.151
Attitudes toward childlessness	.455	.048	.529	9.389	< .001	1.422
Value of parenthood	.112	.046	.132	2.428	.016	1.327
How much do you agree with the civil recognition of homosexual unions?	-.183	.123	-.088	-1.492	.137	1.573
How much do you agree with marriage between same sex individuals?	.007	.071	.006	.103	.918	1.506
How much do you agree with recognition of the opportunity for homosexual people, to adopt children?	.129	.073	.112	1.765	.079	1.815

## Discussion

This study sought to analyse the desires and intentions of becoming parents in a sample of gay and lesbian Italian individuals.

Our data revealed that 84.6% of the participants (M = 84.8%, F = 84.3%) reported parenting desires and that 64.6% (M = 62%, F = 67.7%) declared their intention to have a child. These percentages were high but remain in keeping with data observed in previous studies carried out in the United States (Riskind & Patterson, 2010), Portugal (Costa & Bidell, 2017), and Italy (Baiocco & Laghi, 2013; Rossi et al., 2010). The increasing number of gay and lesbian individuals who reported the desire and the intention to have children indicates that, despite the prevailing prejudices and the Italian legislative system failing to protect lesbian and gay parents, gay and lesbian individuals tend to represent themselves as on a path to parenthood. The current study, in contrast with others carried out in Italy (Baiocco et al., 2014; Scandurra et al., 2019), but similar to a recent Portuguese study (Costa & Bidell, 2017), did not report any significant gender differences in parenting desires and intentions among lesbian and gay participants.

In their studies, Baiocco et al. (2013) and Scandurra et al. (2019) found that lesbian women had higher desire and intention than gay men to become a parent. They explained this data focusing on the strong social pressures on Italian women to become mothers, regardless of their sexual orientation. Our results may be interpreted in the light of the strong family-oriented culture present in the country. In Italy, the desire to have children largely depends on the idea that "making a family" is an important value and this might lead young adults participating in this study to emphasize the positive aspects of becoming parents, regardless of their gender. Moreover, the stigmatization of individuals who voluntarily choose not to have children might have played a role in participants expressing desire and intention of becoming parents, regardless of their gender. Research on the perceptions of people without children has showed more negative perceptions of non-parents than of parents, regardless of their gender, with the former who are perceived as psychologically unfulfilled or maladjusted (Ashburn-Nardo, 2017). These findings indicate parenthood more as a social imperative rather than an individual choice independent from a specific socio-cultural context.

Our data also conveyed a significant gap between parenting desires and parenting intentions not only in the female subsample but also in the male subsample. Thus, the desire to have a child did not always result in the actual intention to have children. This reflects the deep contradictions in the Italian cultural, social and political context, which, on the one hand, emphasizes the values of family and parenting but, on the other hand, denies the recognition of these rights to same-sex parents. Due to this social and political context, the decision by gay men or lesbian women to become parents is more difficult than that for heterosexual men and women (Baiocco & Laghi, 2013) and this may give rise to a gap between desires and intentions.

To fully understand these results, we need to focus on the specific barriers for gay and lesbian individuals seeking to have children (Riskind & Patterson, 2010). In fact, although in recent years several countries have enacted legislation to provide political and social recognition of gay and lesbian parenting, and with scientific research having now demonstrated that children born into a same-sex couple parenting project do not run greater risks in their psychosocial developmental outcomes compared with children raised by heterosexual parents (Biblarz & Stacey, 2010; Tasker, 2010), nonetheless, prejudice regarding lesbian and gay parenting continues, often motivated by conservative religious and political beliefs (Lasio, Congiargiu, De Simone, & Serri, 2019).

In Italy, this prejudice still remains very present because different religious and cultural traditions encourage homophobic opinions and sentiments (Baiocco et al., 2013). Previous research, for example, has shown that religious affiliation and strong religiosity represent significant predictors of negative attitudes toward gay men and lesbian women, and particularly toward marriage equality and LGBT families (Costa et al., 2014; Whitley, 2009). These religious-based negative attitudes are susceptible to internalization by gay, lesbian, and bisexual individuals, who may even come to endorse religious arguments claiming they are not fit to be parents (Lasio, Serri, Ibba, & de Oliveira, 2019). Among LGBT individuals, religiosity partially accounts for their self-stigma and endorsement of negative beliefs about gay and lesbian-parented families (Baiocco et al., 2014; Costa & Bidell, 2017).

Regarding individual political positionings, an Italian study on the attitudes of lesbian and gay individuals towards same-sex parenting (Petruccelli et al., 2015) found that left-wing political orientations correlated with a positive evaluation of lesbian and gay parents. Our study also sought to examine what factors might influence both the desire and the decision to have children. We correspondingly considered, in addition to the personal value attributed to parenting, religious practice and political and ideological positioning. However, contrary to our expectations, religious affiliation, religious involvement and political positioning did not return any significant effect on either parenting desires or intentions. Parenting desires and intentions seemed to be positively influenced mainly by negative attitudes towards childlessness and by the value attributed to parenting. Our results conveyed how individuals who would regret not having children and who valued parenthood, reported higher levels of parenting desires and intentions. Moreover, the level of agreement with the adoption of children by same-sex couples positively impacted on parenting desires and intentions.

Indeed, only age returned a significant effect on the intention to become a parent: higher aged participants declared greater intentions over having children. This data is in line with previous research findings (Baiocco & Laghi, 2013), which identified how older LGBT individuals (up to 35 years old) declared stronger parenting intentions; moreover, these findings are congruent with a general trend in Italy to have children when aged over 31 for women and 35 for men (ISTAT, 2012).

### Limitations and Future Research Directions

There is a need to point out some limitations in our study, which do not allow for any generalization of the results. First, we collected our data in Italy and these findings may not be consistent with the realities prevailing in countries where laws and policies are more favourable toward LGBT individuals. We are currently working on a comparison between certain Mediterranean countries with different political and cultural heritages (Italy, Spain and Portugal) in order to analyse the role that context exerts on the desires and intentions to have children amongst gay and lesbian individuals.

A further limitation of this study stems from having applied only self-report instruments to detect the significantly relevant variables. Future research should also consider recourse to qualitative methodologies (e.g., in-depth interviews) in order to overcome single-method biases and some of the major limitations of the self-report methodology.

Finally, parenting desires and intentions might be influenced by a number of different variables beyond the scope of our study. Our results, for example, might be mediated or moderated by specific factors, such as personal attitudes, family values, motives towards parenthood and the level of social support available. These issues need to be explored in future research.
